# Spinal epidural abscess as predicting factor for the necessity of early surgical intervention in patients with pyogenic spondylitis

**DOI:** 10.1186/s12891-023-06703-4

**Published:** 2023-07-18

**Authors:** Jiwon Park, Sangsoo Han, Yeong Jeon, Jae-Young Hong

**Affiliations:** 1 Department of Orthopedics, Korea University Ansan Hospital, Ansan, 123, Jeokgeum-ro, Danwon-gu, 15355 Ansan-si, Gyeonggi-do Republic of Korea; 2 Department of Emergency Medicine, Soonchunhyang University Bucheon Hospital, 170 Jomaru- ro, 14584 Bucheon, Gyeonggi-do Republic of Korea

**Keywords:** Pyogenic spondylitis, Epidural abscess, Infectious spondylitis, Culture-negative infection

## Abstract

**Background:**

Pyogenic spondylitis is a condition with low incidence that can lead to neurological sequelae and even life-threatening conditions. While conservative methods, including antibiotics and bracing, are considered the first-line treatment option for pyogenic spondylitis, it is important to identify patients who require early surgical intervention to prevent progressive neurologic deficits or deterioration of the systemic condition. Surgical treatment should be considered in patients with progressive neurologic deficits or deteriorating systemic condition. However, currently, there is a lack of treatment guidelines, particularly with respect to whether surgical treatment is necessary for pyogenic spondylitis. This study aims to analyze the radiological epidural abscess on MRI and clinical factors to predict the need for early surgical intervention in patients with pyogenic spondylitis and provide comprehensive insight into the necessity of early surgical intervention in these patients.

**Methods:**

This study retrospectively reviewed 47 patients with pyogenic spondylitis including spondylodiscitis, vertebral osteomyelitis, epidural abscess, and/or psoas abscess. All patients received plain radiographs, and a gadolinium-enhanced magnetic resonance imaging (MRI) scan. All patients have either tissue biopsies and/or blood cultures for the diagnosis of a pathogen. Demographic data, laboratory tests, and clinical predisposing factors including comorbidities and concurrent other infections were analyzed.

**Results:**

We analyzed 47 patients, 25 of whom were female, with a mean age of 70,7 years. MRI revealed that 26 of 47 patients had epidural abscesses. The surgical group had a significantly higher incidence of epidural abscess than the non-surgical group (p = 0.001). In addition, both CRP and initial body temperature (BT) were substantially higher in the surgical group compared to the non-surgical group. There was no significant difference between the surgical group and the non-surgical group in terms of age, gender, comorbidities, and concurrent infectious disorders, as well as the number of affected segments and affected spine levels. However, the surgical group had lengthier hospital stays and received more antibiotics.

**Conclusion:**

The presence of an epidural abscess on MRI should be regarded crucial in the decision-making process for early surgical treatment in patients with pyogenic spondylitis in order to improve clinical outcomes.

**Supplementary Information:**

The online version contains supplementary material available at 10.1186/s12891-023-06703-4.

## Background

Pyogenic spondylitis encompass a diverse spectrum clinical conditions such as pyogenic spondylodiscitis, vertebral osteomyelitis, and epidural and paravertebral abscesses. Despite its low incidence, pyogenic spondylitis can lead to neurological consequences and potentially fatal clinical outcome due to its detrimental impact on the spine and paraspinal structures [1, 2].

Pyogenic spondylitis can be treated conservatively in numerous cases due to the progress made in medical treatment with antibitics. The most crucial step toward achieving effective conservative treatment, is obtaining a microbiologic diagnosis. Notwithstanding the diverse endeavors, in some patients, etiologic organisms are not identified, and only empirical antibiotics are chosen for therapeutic intervention [3–5]. This may elevate the failure of the conservative treatment for pyogenic spondylitis. Challenges in management of the pyogenic spondylitis stem from factors such as delayed microbiologic diagnosis, mechanical instability, and infection by antibiotic-resistant bacteria. In those cases, surgical treatment should be considered in several cases, despite the prolonged treatment with antibiotics.

However, it is questionable whether conservative treatment using antibiotics is adequate or when surgical treatment may be necessary [6–8]. In many institutes such as our hospital, in the form of complete bed rest, intravenous antibiotics, and a spinal brace, conservative treatment is considered an initial treatment in patients with pyogenic spondylitis on their first appearance in hospitals. However, surgical treatment in the form of laminectomy and decompression, debridement, and open biopsy is strongly considered if the patients experience progressive neurologic deficits or deterioration of systemic conditions [1, 3, 6–8].

In this study, we aimed to report the incidence, demographic, clinical, and radiologic characteristics of spontaneous pyogenic spondylitis in 47 patients, as well as to identify the factors that contribute to early surgical intervention in patients with this condition.

## Materials and methods

### Study population

This retrospective study has been approved by the Institutional Review Board of our hospital (2021AS0327). We retrospectively collected medical records of patients over 18 years with suspicion of a diagnosis of pyogenic spondylitis between 2015 and 2020 at a single tertiary hospital. The diagnosis of pyogenic spondylitis was made by infectious disease specialists in conjunction with clinical symptoms, radiologic findings, blood and tissue cultures, laboratory studies, and histological findings. A careful physical examination was conducted by an orthopedic spine surgeon to determine the existence of any neurologic deterioration which required immediate surgical treatment. The inclusion criteria included patients who were (1) diagnosed with pyogenic spondylitis by infectious disease specialists, (2) without deterioration of neurologic function, especially motor function at the time of diagnosis requiring immediate surgical treatment, (3) provided appropriate clinical and radiologic data, including contrast-enhanced MRI, (4) underwent blood culture and tissue culture after admission and before administration of intravenous antibiotics. The exclusion criteria included patients with infectious spondylitis caused by *Brucella* species, *M. tuberculosis*, or fungi polymicrobial infection. Finally, we included 47 consecutive pyogenic spondylitis patients in this retrospective study. Following a retrospective analysis of patient records, the cohort was stratified into two distinct groups: those who received conservative treatment and those who underwent surgical intervention.

### Study measurements

We collected demographic data including sex and age, co-morbidities including diabetes, hypertension, cardiovascular disease, pulmonary disease, chronic kidney diseases and liver diseases, and initial presenting symptoms including pain and/or motor weakness in patients with pyogenic spondylitis. In addition, concomitant infectious diseases including urinary tract infection, upper respiratory infection, pneumoniae, gastrointestinal infection, and endocarditis were recorded.

Patient’s microbiologic diagnosis based on blood and/or tissue biopsy culture were reviewed. Laboratory findings including initial and final white blood cell (WBC) counts, C-reactive protein (CRP), segment neutrophil count (% Seg.) were collected. Body temperature (BT) at the time of admission was recorded.

Radiologically, any instability in infected vertebral segments was identified on plain flexion and extension radiographs. The site of infection (cervical, thoracic, lumbosacral), the number of affected segments, the presence of epidural abscess, and the presence of paravertebral abscess were recorded based on contrast-enhanced MRI.

### Clinical outcomes

The evaluation of changes in motor grade and visual analog scale (VAS) for back and leg pain were conducted to determine the clinical outcome and progression of neurologic deterioration during treatment. Successful treatment of pyogenic spondylitis involves improvement of clinical symptoms, normalization of laboratory findings (with a focus on CRP and WBC), and radiologic improvement on MRI, specifically a reduction in the extent of an epidural or psoas abscess.

### Treatment protocol in patients with suspicious pyogenic spondylitis

In our hospital, infectious disease specialists and/or spine surgeons diagnosed pyogenic spondylitis based on clinical symptoms, radiologic findings, and laboratory studies. Patients exhibiting clinical manifestations indicative of potential pyogenic spondylitis undergo laboratory testing, including WBC, CRP, and % Seg. as well as radiologic examination, which included contrast-enhanced MRI and plain radiographs. The etiologic pathogen was subsequently confirmed through blood culture or a tissue culture by a needle biopsy on the inflame tissues. Since the pathogen usually reach the vertebra or paraspinal tissues through hematogenous spread or during a spinal surgery, procedure, and directly from a site close to the vertebra, a microorganisms cultured from blood is considered as an etiologic pathogen. However, it usually takes time to identify etiologic microorganisms through blood culture or tissue culture.

In our hospital, we usually begin empirical antibiotics based on the most probable microbial etiology in our country. *S. aureus* is the most prevalent microorganism that causes pyogenic spondylitis, although its distribution differs depending on the patient’s baseline medical profile, infection site, and prior spine surgery or treatment. Gram-negative microorganism may be suspected as a etiologic microorganism of pyogenic spondylitis if the patient is female, geriatric, or has a previous or concurrent urinary tract infection or intraabdominal infection.

In our hospital, the selection of the most appropriate empiric antibiotics for patient with pyogenic spondylitis of unknown or not-yet-identified microbial etiology is decided by the infectious disease specialist. If the patient has no history of spinal surgery or treatment, Vancomycin is not the first treatment and excluded from the empiric antibiotics. Instead, first generation of cephalosporin, fluoroquinolone and rifampin, clindamycin, and beta-lactam/beta-lactamase inhibitor are usually administrated. If the patient has a history of urinary tract infection, antibiotics covering gram-negative microorganism is chosen accordingly.

After microbiologic pathogenicity of blood and/or tissue culture is identified, appropriate intravenous antibiotics according to microbial sensitivity and susceptibility are administrated. If the blood and/or tissue culture results reveal negative, empirical antibiotics are continuously administrated until CRP on blood examination becomes normal. We also determine the treatment efficacy based on the cessation of fever and restoration of normal body temperature. We also suggest application of immobilization with a thoraco-lumbo-sacral orthosis (TLSO) to patients to avoid further collapse of vertebral body.

For the treatment of pyogenic spondylitis conservatively, at least six weeks of antibiotics treatment are required. But during this conservative treatment period, surgical treatment is deemed to be required if the patient’s response to conservative treatment is poor, and or are accompanied by progressive neurologic deficit. In our hospital, laboratory studies including WBC, CRP, and % Seg. is performed every 2–3 days for identifying treatment efficacy. During that period, if there is no improvement of laboratory studies or if the patient complains of severe pain or neurologic deterioration, infectious disease specialist consults the orthopedic surgeon asking for the necessity of the surgical treatment.

Surgical treatment is indicated in patients with spinal cord or cauda equine compression with progressive neurologic deficit. Orthopedic spine surgeon carefully evaluates the physical status, especially progression of motor weakness. In addition,obvious spinal instability at the infected vertebral segments developed during the conservative treatment is also indicated for surgical treatment.

Surgical treatments included (1) debridement of infectious tissues through unilateral approach and bilateral decompression surgery; (2)posterior laminectomy and decompression and fusion with or without instrumentation; (3)anterior decompression such as diskectomy or corpectomy and fusion with posterior instrumentation. During the surgery, tissue culture was conducted from both infected segment of vertebral body and adjacent disc materials. The surgical approach depends on the location and severity of pyogenic spondylitis at the moment of surgery.

### Statistical evaluation

Collected data were compared among surgery group and non-surgery group using chi-square test and Fisher’s test. The level of statistical significance was set at a 2-tailed *p* < 0.05. Cut off value of continuous variables was calculated using Youden’s Index. Receiver operating characteristic (ROC) curve was created to measure the cut off value of laboratory test results. All statistical analyses were performed using the SAS software (version 9.3; SAS Institute, Cary, NC, USA); *P* values < 0.05 were considered statistically significant.

## Results

### Demographic, clinical, and radiologic characteristics of Study population

A total 47 patients (21 males, 26 females) were retrospectively reviewed. Mean age was 75 years old (64.5–81). Mean hospital stays for treatment was 43 days (33.25–64.75 days), and mean days for antibiotic treatment was 44 days (33.5–66 days). Initial presenting symptoms of the patients was either (1) back pain or lower extremity radiating pain in 31 patients (VAS > 5), or (2) back pain or lower extremity radiating pain with subjective lower extremity weakness (VAS > 5 with normal lower extremity motor grade) in 12 patients. Among 47 patients, 17 patients (37.17%) had diabetes mellitus, and 8 patients (17.02%) had chronic kidney diseases. 29 patients (61.6%) had one or concomitant infection such as urinary tract infection (18/47, 38.3%), gastrointestinal infection (9/47, 19.15%), and pneumoniae (6/47, 12.77%). 14 patients (29.79%) had previous history of spine surgery and 9 patients (19.15%) had previous history of spine injection. The most commonly involved area of pyogenic spondylitis was lumbar spine (27/47, 57.45%) followed by lumbosacral spine (8/47, 17.02%). In 32 patients (68.09%), the number of affected segments was limited in single level. In MRI, epidural abscess was observed in 26 patients (55.32%), and paravertebral abscess was observed in 26 patients (55.32%). (Table 1).

**Table 1:** Demographic and clinical features of the 47 patients with pyogenic spondylitis

Characteristics	
Age (years)	75 (64.5, 81)
Sex	
Male	21
Female	26
Co-morbidity	
Diabetes	17 (36.17%)
Hypertension	25 (53.19%)
Cardiovascular disease	4 (8.51%)
Pulmonary disease	3 (6.38%)
Chronic kidney disease	8 (17.02%)
Liver disease	17 (36.17%)
Concomitant infection	
Urinary tract infection	18 (38.3%)
Upper respiratory infection	2 (4.26%)
Pneumoniae	6 (12.77%)
Gastrointestinal infection	9 (19.15%)
Endocarditis	3 (6.38%)
Other infections	3 (6.38%)
Body temperature at admission	37.65 ± 0.75
Laboratory data	
White blood cell count	9730 (7880, 15,385)
Segmented neutrophil count (%)	80.2 (71.6, 87.45)
C-reactive protein (mg/dL)	12.12 (3.86, 27.4)
Radiologic finding	
Involved levels	
Cervical spine	0
Cervicothoracic spine	6 (12.77%)
Thoracic spine	27 (57.45%)
Thoracolumbar spine	2 (4.26%)
Lumbar spine	4 (8.51%)
Lumbosacral spine	8 (17.02%)
Epidural abscess	26 (55.32%)
Paravertebral abscess	26 (55.32%)
Psoas abscess	12 (25.53%)
Duration of hospital stays (days)	43 (33.25, 64.75)
Duration of antibiotic treatment (days)	3 (2, 3)

### Etiologic microorganisms

Of 47 patients, 28 patients (59.57%) had culture positive results in either blood culture (25/47, 53.19%) or tissue culture (9/47, 19.15%), and culture-negative rate was 45.8% (20/47). Among those 28 patients with identified etiologic microorganisms, 21 patients had gram positive cocci infection. The most common etiologic pathogen was *staphylococcus aureus* (18/28, 64.3%), followed by *streptococcus* species). 6 patients had gram negative infection: *Pseudomonas aeruginosa* (2 patients), *Klebsiella pneumoniae*, *Escherichia coli, Bacteroides fragilis, and Proteus mirabilis.* (Table 2).

**Table 2:** Etiologic microorganism based on blood and/or tissue culture results

Culture negative	19 (49.43%)
Culture positive	28 (59.57%)
Blood culture positive	25 (53.19%)
Tissue culture positive	9 (19.15%)
Etiologic Organisms	
Gram positive	22 (46.8%)
Staphylococcus	19 (40.43%)
Streptococcus	3 (6.34%)
Gram negative	6 (12.67%)
Pseudomonas aeruginosa	2
Klebsiella pneumoniae	1
Escherichia coli	1
Bacteroides fragilis	1
Proteus mirabilis	1

### Different characteristics between surgical group versus non-surgical group

The demographic findings and clinical characteristics of surgical group and non-surgical group are shown in Table 3. There were no significant differences in age and sex between two groups (*p =* 0.2967 and *p* = 0.8957, respectively). With respect of co-morbidities such as diabetes and chronic kidney diseases, there were no significant differences in co-morbidities. In addition, two groups had no statistical differences in concomitant infectious disorders such as urinary tract infection, gastrointestinal infection, endocarditis and pneumoniae.

**Table 3:** Comparison of clinical and radiologic characteristics of non-surgical group versus surgical groups

Characteristics	Non-Surgical group (n = 24)	Surgical group (n = 23)	P-value
Age (years)	78 (64.25, 84.25)	74 (66.5, 80)	0.2967
Sex			
Male	10 (41.67%)	11 (47.83%)	0.8957
Female	14 (58.33%)	12 (52.17%)	
Co-morbidity			
Diabetes	9 (37.5%)	8 (34.78%)	> 0.99
Hypertension	12 (50%)	13 (56.52%)	0.8764
Cardiovascular disease	1 (4.17%)	3 (13.04%)	0.3475
Pulmonary disease	1 (4.17%)	2 (8.7%)	0.6085
Chronic kidney disease	4 (16.67%)	4 (17.39%)	> 0.99
Liver disease	9 (37.5%)	8 (34.78%)	> 0.99
Concomitant infection	13 (54.17%)	16 (69.57%)	0.4322
Urinary tract infection	9 (37.5%)	9 (39.13%)	> 0.99
Upper respiratory infection	1 (4.17%)	1 (4.35%)	> 0.99
Pneumoniae	3 (12.5%)	3 (13.04%)	> 0.99
Gastrointestinal infection	3 (12.5%)	6 (26.09%)	0.2865
Endocarditis	2 (8.33%)	1 (4.35%)	> 0.99
Other infections	1 (4.17%)	2 (8.7%)	0.6085
Body temperature at admission	37.38 ± 0.65	37.93 ± 0.75	**0.0101**
Laboratory data			
White blood cell count	9150 (7042.5, 11232.5)	11,800 (8140, 18,000)	0.0515
Segmented neutrophil count (%)	75.4 (70.03, 86.43)	84 (76.1, 88.35)	0.2054
C-reactive protein (mg/dL)	6.91 (2.86, 17.37)	14.23 (7.78, 28.24)	0.1039
Radiologic finding			
Epidural abscess	5 (20.83%)	21 (91.3%)	**< 0.001**
Paravertebral abscess	10 (41.67%)	16 (69.57%)	0.1032
Psoas abscess	6 (25%)	6 (26.09%)	> 0.99
Duration of hospital stays (days)	34 (18, 45)	56 (40.5, 83.5)	0.0011
Duration of antibiotic treatment (days)	35 (20.5, 51)	55 (42.5, 85.5)	**0.0025**
Recurrence	4 (16.67%)	4 (17.39%)	> 0.99

Laboratory findings including WBC, CRP (mg/dL) and % Seg. at admission were not significantly different between two groups although there were tendencies of increasing WBC, CRP and % Seg. in surgical group.(WBC: 9,150 (7042.5 ~ 11232.5 vs. 11,800 (8140 ~ 18,000); CRP 6.91 (2.86–17.37) vs. 14.23 (7.78–28.24); and % Seg. 75.4 (70.03–86.43) VS. 84 (76.1-88.35); non-surgery group vs. surgery group, respectively) Initial BT was slightly higher in surgical group with statistical significance (*p* = 0.0101). There was no significant difference in initial presenting symptoms between two groups. With respect of MRI findings, there was significantly more patients with epidural abscess in surgical group than non-surgical group (21 patients versus 5 patients, *p* = 0.0001). There was no significantly difference in paravertebral abscess as well as psoas abscess (*p* = 0.1032 and *p* > 0.99, respectively). Both surgical group and non-surgical group had pyogenic spondylitis most commonly in lumbar spine (54.17% and 60.87%). There was no significant difference in culture positive rate between two groups. While, there was a higher rate of *Staphylococcus* infection in surgical group even though it was not statistically significant (52.17% versus 29.17%).

Total length of hospital stays was significantly longer in surgical group (56 days versus 34 days, *p* = 0.0011). Surgical group had longer duration of antibiotic treatment than non-surgical group (55 days versus 35 days, *p* = 0.0025). However, there was no significant difference in recurrence rate between two groups. Regardless of surgery, most patients in both surgical group and non-surgical group showed improvement in symptoms after treatments (86.96% and 78.26%). (Table 3) In our study, symptomatic improvement was regarded as possible discharge without neurologic deterioration.

### Cut off value of laboratory testing results for consideration of surgical treatment

Table 4 showed cut off value of continuous laboratory testing results as well as BT. Cut off value for consideration of surgical treatment was (1) WBC with 11,080; (2) CRP with 5.61 mg/L; (3) % Seg. with 75.4%. In Table 5, we also calculated the BT cut off value of 37.05º. With multivariable logistic regression model, we found that epidural abscess had odds ratio of 27.49 (95% confidence interval of 4.49 ~ 168.23, *p* = 0.0003).

**Table 4:** Univariate regression model for identifying risk factors for surgery

	Odds ratio	Lower bound of 95% CI for Odds ratio^*^	Upper bound of 95% CI for Odds ratio^*^	P-value
WBC at admission	1.0001	1	1.0002	0.043
CRP at admission	1.0318	0.9826	1.0834	0.209
%Seg at admission	1.0457	0.9922	1.1021	0.0956
WBC_Cut_11080	3.6833	1.0624	12.7705	**0.0398**
CRP_cut_5.61 (mg/dL)	7.2727	1.683	31.4266	**0.0079**
%Seg_cut_76.35 (%)	3.6833	1.0624	12.7705	**0.0398**
BT at admission	3.5422	1.3432	9.3411	0.0106
BT_cut_37.05 (°)	7.5	1.4232	39.5232	**0.0175**
Staphylococcus infection	2.6494	0.7966	8.8112	0.112
Concomitant infection	1.9341	0.5841	6.4042	0.2802
Epidural abscess	37.8	6.5266	218.9249	**5.051 × 10** ^**− 05**^

**Table 5:** Multivariate regression model for identifying risk factor for surgery

	Odds ratio	Lower bound of 95% CI for odds ratio		Upper bound of 95% CI for odds ratio	P-value
CRP_cut_5.61 (mg/Dl)	2.7435	0.3866		19.4685	0.3128
BT_cut_37.05 (°)	3.489		0.3929	30.9855	0.2621
epidural_abscess1	27.4879		4.4914	168.2286	**0.0003**

## Discussion

Conservative treatment of pyogenic spondylitis has become a viable option in many cases, thanks to the advancements in medical treatment utilizing antibiotics. However, it is questionable whether conservative treatment using antibiotics is adequate or as to when surgical treatment may be necessary. In cases where patients exhibit a decline in systemic conditions or progressive neurologic deficits, surgical intervention is highly recommended. In the present research, we aimed to report the prognostic factors that contribute to early surgical treatment in patients with this condition.

In our study, the most significant prognostic factor associated with early surgical treatment was the presence of a spinal epidural abscess on MRI, regardless of its location and degree of dural sac compression. The present study exclusively enrolled patients who exhibited initial symptoms of back pain and/or radiating pain in the lower extremities, but did not display any objective motor weakness. It was not deemed necessary to perform surgical intervention on all participants in the study. Surgical treatment was advised for patients with pyogenic spondylitis who experienced progressive neurological decline or inadequate relief of laboratory test results despite receiving adequate intravenous antibiotic therapy for a sufficient duration. When comparing the surgical group and the non-surgical group, we found that epidural abscess on initial MRI regardless of dural sac compression was strongly associated with the surgical treatment (odds ratio of 27.49 with 95% confidence interval for 4.49–168.23, *p* = 0.0003). Even though there are no guidelines for surgical treatment of pyogenic spondylitis, several studies favor early surgical treatment for spinal epidural abscess, and our results support the previously reported conclusions [15–18].

Spinal epidural abcess an infrequent yet severe ailment that arises from an accumulation of purulent fluid in the space between the spinal dural mater and the vertebral periosteum. The current approach for managing this condition, particularly when neurological deficits are present, involves performing surgical decompression and evacuation alongside antibiotic treatment. Prior research has indicated that therapeutic interventions may have advantageous effects on the neurological state of the patients. In addition to the commonly accepted surgical indications for pyogenic spondylitis, including neurologic decline, mechanical instability, and persistent pain or unsuccessful conservative treatment with prolonged antibiotics, we suggest that surgical treatment be considered promptly upon diagnosis of epidural abscess on MRI to prevent further neurological deterioration. Patel et al. found that patients who received surgical treatment following an unsuccessful conservative antibiotics treatment exhibited compromised motor function recovery, despite the surgical intervention [16]. According to Ghobrial et al., early surgical intervention for spinal epidural abscess was found to be advantageous for all patients who exhibited neurological deterioration, although statistical significance was not observed [18].

We also found that higher BT at admission was strong predictor for the failure of conservative treatment and for the necessity of surgical treatment. Fever is one of two most common clinical features in patients with pyogenic spondylitis with spinal pain, and these are the sole symptoms that are recognized to manifest prior to the decline of neurological condition [19]. Also, according to Chang et al., less than 50% of all patients exhibited fever, contrary to expectations [20]. In our study, patients who underwent surgical treatment exhibited higher BT at admission (37.93 ± 0.75 ºC) compared to the non-surgical group (37.38 ± 0.65 ºC) (*p* = 0.0101). We also found that BT cut off value for the necessity of surgical treatment was 37.05ºC at admission (p = 0.0175 in univariate regression model, and p = 0.003 in multivariate regression model). Therefore, when infectious disease specialist first exams the patients suspicious of pyogenic spondylitis, fever serves as a significant indicator for assessing the possibility of progression of pyogenic spondylitis when used in conjunction with laboratory analyses and MRI results,

The laboratory test results of our study indicate that the surgery group exhibited elevated levels of WBC count, CRP and segmented neutrophil counts in comparison to the non-surgery group although the observed differences were not statistically significant. Our calculated cut off value of CRP for the necessity of surgical treatment was 5.61 (mg/dL) (*p* = 0.0079 in univariate regression analysis). Nonetheless, elevated WBC as well as elevated CRP were found in our study, and this result is consistent with other studies [15, 16]. Pyogenic spondylitis is caused by hematogenous spread of etiologic microorganism and is usually treated conservatively with antibiotics [4, 9−11]. Therefore, as mentioned previously, the essential element for successful conservative treatment is the diagnosis of the etiologic pathogen [1, 4, 21]. It usually take 2–3 days to identify etiologic microorganism via blood culture. However, the rate of successful identification varies, and some studies reported lower culture-positive rate with less than 50% [5, 11–13, 21–22]. This might be the factors affecting the success of conservative treatment.

The microorganisms responsible for pyogenic spondylitis exhibit a wide range of diversity, and their distribution may vary regionally and periodically. Therefore, it is important to understand the etiology and microbiology in each patient prior to start conservative treatment using empirical antibiotics [21–22]. It is well accepted that various predisposing factors such as diabetes mellitus, age, injecting drug use, immunosuppression, malignancy, renal failure, liver cirrhosis and spinal surgery are closely associated with a specific microorganism for infection [21].

Our study showed culture-positive rate with 59.57% with the predominant pathogen of *Staphylococcus aureus*, and this result is consistent with the previously reported studies, that *S. aureus* is predominantly characterized as a primary causative microorganism [10, 20−21]. Gram negative bacterium account for 7 ~ 33% of pyogenic spondylitis cases according to previous study, and this result are consistent with ours: 12.67% of gram-negative microorganism in the culture-positive patients [21]. In our hospital, in order to determine the optimal empirical antibiotics for a patient with pyogenic spondylitis of unknown origin, we first consider various predisposing factors first. We suggest to use first generation of cephalosporin, fluoroquinolone and rifampin, clindamycin, and beta-lactam/beta-lactamase inhibitor as a first line empirical treatment if the patient has never undergone a spinal surgery or procedures. For patients included in our study, empirical antibiotics covering *Staphylococcus aureus* and *Escherichia coli* were administrated empirically in all culture-negative patients.

In our study, approximately half of the patients (23/47) underwent surgical treatments. The duration of intravenous antibiotics was 35 days (20.5–51 days) for the conservatively treated patients (non-surgical group), and even though this 5 week of antibiotics administration was slightly shorter than the recommended treatment of 6 weeks in previous studies, most of patients (83.33%) had been treated successfully without relapse. In surgery group, significantly more duration of hospital stay and intravenous antibiotic treatment (35 and 34 days in non-surgery group vs. 36 and 55 days in surgery group, *p =* 0.0011 and p = 0.0025, respectively). We assumed that the treatment period in the surgery group took longer than in the non-surgery group because (1) there was no effect of conservative treatment until surgical treatment, (2) patients whose condition worsened due to neurologic deterioration received surgical treatment, and (3) additional antibiotic treatment was performed for at least 5–6 weeks from the time of surgical treatment. Therefore, it is important for infectious disease specialist as well as spine surgeon to predict when surgical treatment will be needed and to decide on surgical treatment.

Interestingly, there was no significant difference in co-morbidities such as diabetes mellitus and chronic kidney diseases between surgical group and non-surgical group. Although diabetes mellitus has been reported one of the predict factors for conservative antibiotics treatment, our study did not yield same results [16]. This might be due to small size of study population in our study. As other previous studies have emphasized, it is important to determine the predisposing risk factors of pyogenic spondylitis, use appropriate antibiotics accordingly, and determine surgical treatment based on MRI findings. Based on our study, we suggest that initial presenting BT, and CRP as well as existence of epidural abscess can be a significant risk factor for predicting failure of conservative treatment and necessity of surgical treatment.

Our study has several limitations. First, our study is a retrospective case series with relatively small study populations. This may have resulted in an underpowered analysis. Secondly, we were unable to include the degree of spinal cord compression and extent of the epidural abscess on MRI in this analysis. However, we will certainly consider incorporating these parameters in this study, as we recognize the importance of radiological assessment in evaluating the severity and progression of spinal epidural abscess. Thirdly, we did not evaluate the duration of intravenous antibiotics treatment before and after surgical treatment. Therefore, failure of antibiotics treatment and the assessment of the response to surgical treatment could not be accurately identified. Lastly, analysis of antibiotic-resistant organism is important to understand the effect of prolonged antibiotic usage, but in this study, we were not able to analyze resistant organisms in our current study. To overcome these limitation, further studies with a larger number of study population should be conducted, and we will do further study regarding pyogenic spondylitis.

In conclusion, radiologically identifying epidural abscess regardless of neurologic symptoms is the strong risk factor for surgical treatment for pyogenic spondylitis. In addition, surgical treatment should be considered as early when patient presents initially elevated CRP and high BT with epidural abscess for the better clinical outcome for pyogenic spondylitis.

## Electronic supplementary material

Below is the link to the electronic supplementary material.

Supplementary Material 1

Supplementary Material 2

## Data Availability

Data is available via a request to the corresponding author.

## List of abbreviations

MRI: Magnetic resonance images
WBC: white blood cell
CRP: C-reactive protein
%Seg: segment neutrophil count
BT: body temperature
TLSO: thoraco-lumbo-sacral orthosis
ROC: receiver operating characteristic
