# Supplementary material for: Spinal epidural abscess as predicting factor for the necessity of early surgical intervention in patients with pyogenic spondylitis
Source: BMC Musculoskelet Disord. 2023 Jul 18;24:586. doi: 10.1186/s12891-023-06703-4 (PMC10355007; doi:10.1186/s12891-023-06703-4)
Supplement: Supplementary file 1 — Supplementary Material 1 [file 12891_2023_6703_MOESM1_ESM.docx]

**Supplementary Figure Legneds**

sFigure 1. Receiver operating characteristics curve for (A) CRP, (B) BT, and (C) epidural abscess
