# Supplementary figures and images for: Spinal epidural abscess as predicting factor for the necessity of early surgical intervention in patients with pyogenic spondylitis
Source: BMC Musculoskelet Disord. 2023 Jul 18;24:586. doi: 10.1186/s12891-023-06703-4 (PMC10355007; doi:10.1186/s12891-023-06703-4)

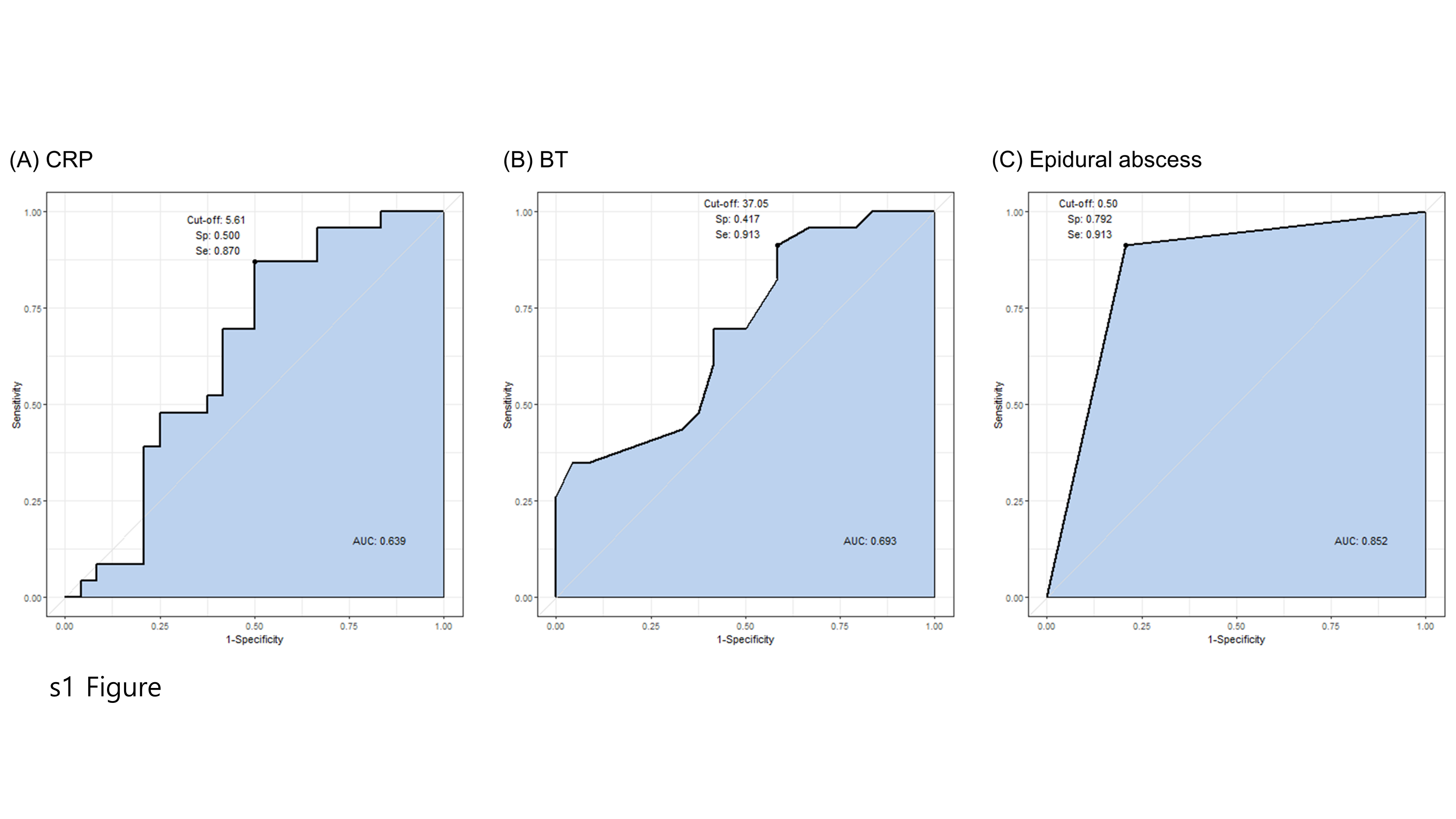

Supplement: Supplementary file 2 — Supplementary Material 2 [file 12891_2023_6703_MOESM2_ESM.png]
